# The propofol-sparing effect of intravenous lidocaine in elderly patients undergoing colonoscopy: a randomized, double-blinded, controlled study

**DOI:** 10.1186/s12871-020-01049-z

**Published:** 2020-05-30

**Authors:** Mengmeng Chen, Yi Lu, Haoran Liu, Qingxia Fu, Jun Li, Junzheng Wu, Wangning Shangguan

**Affiliations:** 1 Department of Anesthesiology and Perioperative Medicine, The Second Affiliated Hospital and Yuying Children’s Hospital of Wenzhou Medical University, 109 West Xueyuan Road, Wenzhou, 325027 China; 2 Department of Anesthesia and Pediatrics, Cincinnati Children Hospital Medical Center, Cincinnati, OH USA

**Keywords:** Anesthetic, Colonoscopy, Endoscopy, Lidocaine, Propofol

## Abstract

**Background:**

Propofol provides a prominent sedation effect in colonoscopy. However, anesthesia and sedation induced with propofol in the elderly might result in cardiopulmonary complications, especially when it is combined with opoids in the regimen. This study aimed to test the hypothesis that the addition of intravenous lidocaine to propofol-based sedation could decrease the overall propofol requirement in elderly patients during colonoscopy while the procedural sedation satisfaction and the hemodynamic stability were not compromised.

**Methods:**

Ninety-two patients undergoing colonoscopy were randomly enrolled into lidocaine+propofol (L + P) group or normal saline+propofol (NS + P) groups. Subjects received intravenous bolus of 1.5 mg/kg lidocaine followed by 4 mg kg^− 1^ h^− 1^ lidocaine continuous infusion in L + P group or equivalent volumes of normal saline for boluses and infusion in NS + P group. Anesthesia was induced with 2.5 μg sufentanil followed by injection of 1.2 mg kg^− 1^ propofol in all patients. A single supplemental bolus of 0.6 mg kg^− 1^ propofol was administered whenever MOAA/S score > 1 or had body movement during the colonoscopy. The recorded primary endpoints included: the total amount of propofol administered during entire procedure, the supplemental amount of propofol after induction, and the frequencies of boluses of supplemental propofol.

**Results:**

A total of 79 patients were included in the final analysis. Compared with NS + P group, the total amounts of propofol (induction plus supplemental) were no significant differences in L + P group; however, the required supplemental propofol was less (69.9 ± 39.2 mg vs. 51.5 ± 38.6 mg) (*P* = 0.039); the average frequencies of boluses of supplemental propofol given after induction were lower (2.1 ± 1.1 vs. 1.4 ± 0.9) (*P* = 0.003); the calculated “unit propofol” infusion rate was lower (0.18 ± 0.05 vs. 0.14 ± 0.04 mg kg^− 1^ min^− 1^) (*P* = 0.002).

**Conclusions:**

The addition of intravenous lidocaine to propofol-based sedation resulted in a remarked reduction of supplemental propofol in the elderly during colonoscopy.

**Trial registration:**

The present clinical trial was registered at http://www.chictr.org.cn on 11th March 2019 (registration No. ChiCTR1900021818).

## Background

The incidence of colorectal cancer in elderly patients is rising in developing countries including China [1, 2]. Colonoscopy has been considered as the gold standard approach to provide the preventive measure and early detection for colorectal cancer, in which the suspicious polyps and adenomas in the colorectum can be removed and the pathologic diagnosis can be confirmed [3, 4].

Sedation and anesthesia are frequently applied during colonoscopy to reduce patients’ discomfort [5–7]. Midazolam, or fentanyl, alone or combined has been the mainstream of sedative drugs used for this procedure over the years. More concerned side effects for midazolam are drowsiness afterwards, and paradoxical reaction etc. [8, 9]. Dexmedetomidine has also been tried, but only showed a limited role in this setting for its slow onset, tedious preparation of intravenous infusion, and longer recovery time and delayed discharge [10], and even a low-dose dexmedetomidine with propofol has been confirmed to delay discharge readiness and provoke hypotension compared to propofol alone [11]. Propofol has been getting more popular as sedative during colonoscopy for its advantages of fast onset and offset, quick recovery and less perception of procedure pain and discomfort during the colonoscopy with higher patient’s satisfaction [12, 13]. The majority of colonoscopic procedures are performed in outpatient clinic setting and patients are discharged quickly at the end of recovery, and therefore, propofol is the most optimal drug for the same day surgeries. Recent years, propofol sedation has been proved safe and practical for outpatient colonoscopy over 20 years old [14]. Propofol alone as a sedative in colonoscopy may require a larger dose to maintain a sufficient depth of anesthesia and supplement with low-dose opioids could reduce the stimulation on autonomic nerves system caused by colonoscopy [15], and make the patient recovery faster. Opioids sedatives have been widely used in enteroscopy [16]. Fahima et al. [17] found that combined administration of opioids with propofol in colonoscopy was statistically safe to the patients with higher satisfaction feedback. However, the increased sensitivity to propofol in some individuals, especially in elderly patients could lead to the development of profound hypotension and prolonged apnea when propofol is used as a sedative during colonoscopy.

Lidocaine is a short-acting local anesthetic and intravenous lidocaine has been shown to have analgesic and sedative effect. Therefore, administration of lidocaine combined with propofol has been used as a strategy to decrease the overall propofol requirement of during sedation [7]. Other perioperative benefits of intravenous lidocaine include lower fatigue rate, shorter hospital stays [18, 19] and the reduction of propofol injection pain at peripheral intravenous site and the less demand for anesthetic [7, 20]. However, to the best of our knowledge, the study concerning about propofol-sparing effect of intravenous lidocaine in the elderly patients undergoing colonoscopy has not been reported in the literature. The goal of our investigation was to find out whether the addition of intravenous lidocaine to propofol-based sedation would reduce the overall propofol requirement and, at the same time, reserve the hemodynamic and respiratory normality in elderly population during colonoscopy.

## Methods

This study was reviewed and approved by the Ethics Committee of the Second Affiliated Hospital of Wenzhou Medical University (No. LCKY2019–09) and was registered in www.chictr.org.cn (registration number ChiCTR1900021818). This study adhered to the applicable CONSORT guidelines. According to relevant literature reports, it has been reproted that a sample size of 18 patients per group would provide an 80% power for detecting a 30% difference in propofol needs between groups at an alpha level of 0.05 [7]. The power was raised to 90% instead of 80% in this study, on the basis of a 0.05 alpha level and a 90% power for detecting a 30% difference in propofol needs between groups, according to the data from reference 7, the value [200(109) vs. 128(53)] were put into the sample size calculation formula (*n* = 2*[(α + β) σ/δ]^2). Then a sample size of 30 patients per group were calculated and a total of 60 patients were finally included. The number of cases falling off during the experiment was calculated as 35%, so the final sample size was 92 cases. After informed consent was obtained, 92 patients, aged ≥65, ASA I-II, undergoing colonoscopy under sedation were initially included in this randomized, double-blind, controlled clinical study. Exclusion criteria included: severe cardiovascular and pulmonary diseases such as hypertension and respiratory insufficiency; mental disorders, such as schizophrenia and psychosis on long-term psychotropic drugs; history of having previous colectomy; hyperalgesia or refractory cancer pain; and intravenous anesthesia contraindication. The study began to recruit patients on March 11, 2019, and the first case enrolled was on March 12, 2019. The last patient completed on September 25, 2019, and the experiment ended. All patients participating in this study did not receive serious accidental harm.

The sequential numbers from 1 to 92 for patient enrollments were marked outside of each individual envelopes, in which 46 paper slips marked with #1 [Lidocaine+Propofol (L + P) group] and 46 slips marked with #2 [Normal saline+Propofol (NS + P) group] were placed inside based on a computer-generated randomized order, and then the envelopes were sealed. On the day of study, an envelope with the smallest sequential number was opened first and the solutions of medicines were prepared in a syringe by an investigator based on the # number appeared on the paper slip, either #1 or #2. Then, the unlabeled syringes of solutions were handed over to an anesthesiologist who was performing general anesthesia and administrating the medicines, but were blind to the contents inside of the syringes. Another anesthesiologist who observed and recorded the data intraoperatively and postoperatively was also blinded to the medication patient had received. All sedation procedures were standardized and performed by the same anesthesiologist who was blind to the patient groups. Patients were divided into two groups: L + P group and NS + P group according to the random assignment generated by an anesthesiologist through computer. Prior to anesthesia induction, the standard monitors of heart rate, non-invasive blood pressure and pulse oximeter were placed. Patients in L + P group received intravenous bolus of 1.5 mg kg^− 1^ lidocaine over 10 s (2% lidocaine) followed by 4 mg kg^− 1^ h^− 1^ continuous lidocaine infusion (0.5% lidocaine), and patients in NS + P group were given the equivalent volumes of normal saline in boluses and infusion. Then, all subjects in both groups were induced with 2.5 μg sufentanil and 1.2 mg kg^− 1^ propofol intravenously. Patients were kept breathing spontaneously over venture mask with oxygen flow at 6 L min^− 1^. All patients were told to feedback to the anesthesiologist if injection pain happened during the initial propofol administration. The level of sedation was assessed every minute with MOAA/S scale (Modified Observer’s Assessment of Alertness/Sedation Scale: Level 5: Responds readily to name spoken in normal tone; Level 4: Lethargic response to name spoken in normal tone; Level 3: Responds only after name is called loudly and/or repeatedly; Level 2: Responds only after mild prodding or shaking; Level 1: Responds only after painful trapezius squeeze; Level 0: No response after painful trapezius squeeze). The colonoscope was inserted by endoscopist after the patients’ MOAA/S score was ≤1. To maintain an adequate sedation level after induction, a supplemental bolus of 0.6 mg kg^− 1^ propofol was administered once MOAA/S score > level 1 and this step could be repeated as needed. All meditation stopped after the endoscopy completed. The primary endpoints included: the total amount of propofol administered during the procedure, the supplemental amount of propofol after induction, and the frequencies of boluses of supplemental propofol. The secondary endpoints included: values of non-invasive blood pressure (MAP), and oxygen saturation (SpO_2_) at different time points during colonocropy; propofol injection pain; the occurrence of adverse events. All the data was colletcted in the Second Affiliated Hospital of Wenzhou Medical University.

Data were analyzed with the SPSS 20.0 (SPSS Inc., Chicago, IL, USA). Normally distributed data were expressed as mean ± standard deviation and the data that did not conform to the normal distribution were expressed as p_50_ (p_25_, p_75_). Sex was compared using Chi square test. Age, body weight between the two groups were compared using study t-test. MAP, SpO_2_, endoscopic examination time and recovery time between two groups were compared using study t-test. MAP, SpO_2_ at diffirent time points within the group were compared with one-way ANOVA and Bonferroin correction. The incidence of propofol injection pain was compared using Chi square test. The incidence of apena was compared using Chi square test. The total amount of propofol (induction and supplemental) was compared with Wilcoxon rank sum test, and then, the “unit propofol”, defined as mg kg^− 1^ min^− 1^ was calculated by factoring the patients’ weight and the length of anesthesia into the total amount of consumed propofol, and compared between groups using t-test. The total of supplemental propofol (mg) and the frequency of supplemental boluses of propofol being administered after the initial dosage of anesthesia induction were compared using t-test. *P* value less than 0.05 was considered statistically significant.

## Results

**Fig. 1:**
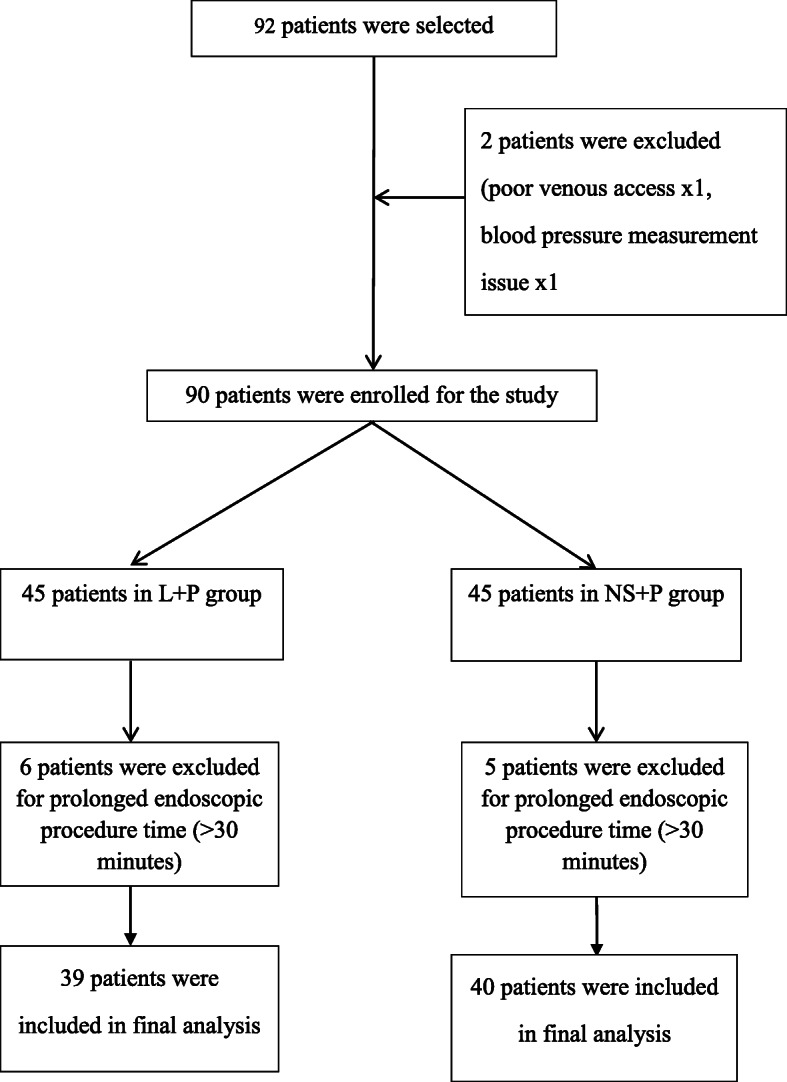
Flowchart of the study

92 patients were initially enrolled and 79 patients completed this study, detailed in Fig. 1. There were no significant differences in general characteristics such as age, sex, and body weight between the two groups (Table 1).

**Table 1:** The general characteristic data in the two groups

	NS + P group (*n* = 40)	L + P group (*n* = 39)
Age (yr)	71.4 ± 5.1	70.4 ± 4.5
Gender (M/F)	13/27	22/17
Body weight (kg)	57.6 ± 11.5	62.4 ± 12.3
Endoscopic examination time (min)	12.3 ± 4.5	12.3 ± 5.1
Recovery time (min)	4.4 ± 3.0	3.9 ± 3.6
Incidence of IV injection pain (n)	18	11

The endoscopic procedure time and the anesthesia recovery time were no significant differences (Table 1). In terms of hemodynamic profiles, MAP and SpO_2_ did not have significant differences between the two groups; within the group, compared with baseline, MAP was lower at various time points after the anesthesia, while there was no significant difference in SpO_2_ at different time points (Table 2). 2 patients suffered from brief apnea in L + P group and none in NS + P group, but there was no statistical difference between the two groups (*P* = 0.15).

**Table 2:** Comparison of perioperative MAP and SpO_2_ between the two groups

Timelines	NS + P group (*n* = 40)		L + P group (*n* = 39)	
	MAP (mmHg)	SpO_2_ (%)	MAP (mmHg)	SpO_2_ (%)
Baseline	100.5 ± 15.3	98.8 ± 1.5	98.5 ± 13.9	98.7 ± 1.6
Immediately after induction	82.3 ± 14.0^*^	99.0 ± 1.7	82.5 ± 14.7^*^	98.5 ± 1.3
3 min after induction	76.4 ± 14.4^*^	98.7 ± 1.7	77.1 ± 13.2^*^	98.0 ± 2.0
5 min after induction	77.2 ± 13.1^*^	98.6 ± 1.8	77.1 ± 14.0^*^	98.1 ± 2.0
10 min after induction	75.5 ± 12.4^*^	98.5 ± 1.6	78.8 ± 12.7^*^	97.8 ± 2.0
15 min after induction	79.9 ± 9.0^*^	98.3 ± 1.2	74.6 ± 12.9^*^	97.9 ± 1.5
20 min after induction	82.7 ± 5.0^*^	98.5 ± 2.1	69.8 ± 16.9^*^	98.0 ± 0.8
Endoscopy finished	73.0 ± 11.9^*^	98.9 ± 1.3	75.2 ± 11.9^*^	98.4 ± 1.4
Fully awake in recovery room	74.3 ± 13.9^*^	98.2 ± 1.8	79.4 ± 13.3^*^	97.4 ± 2.1

Compared with baseline, ^*^P < 0.005

Overall, the L + P group consumed less total amount of propofol [122.4 (93.0, 162.0), 95% CI (141.7–166.3)] than NS + P group did [136.8 (104.6, 175.8), 95% CI (111.9–138.4)] (*P* = 0.06) during the whole procedure, but the difference was not statistically significant. Totally 32 and 31 patients needed additional bolus in L + P group and NS + P group respectively. The supplemental propofol (mg) propofol given after induction in L + P group [(51.5 ± 38.6), 95% CI (39.4–63.6)] was significant lower than that in NS + P group [(69.9 ± 39.2), 95% CI (57.7–82.1)] (*P* = 0.039). The frequency (times) of supplemental boluses of propofol in L + P group (1.4 ± 0.9) was significant lower than that in NS + P group (2.1 ± 1.1) (*P* = 0.03).

Table 3 showed that the calculated “unit propofol” infusion rates (mg kg^− 1^ min^− 1^) indicated a significant reduction in L + P group (0.14 ± 0.04) compared to NS + P group (0.18 ± 0.05) (*P* = 0.002).

**Table 3:** Comparison of propofol doses between the two groups

Items	NS + P group (*n* = 40)	L + P group (*n* = 39)
Total propofol (mg) (95% CI)	136.8 (104.6, 175.8) (111.9–138.4)	122.4 (93.0, 162.0) (141.7–166.3)
Supplemental propofol (mg) (95% CI)	69.9 ± 39.2 (57.7–82.1)	51.5 ± 38.6^#^ (39.4–63.6)
“Unit propofol” (mg kg^−1^ min^−1^)	0.18 ± 0.05	0.14 ± 0.04^*^
Frequency of supplemental boluses (times)	2.1 ± 1.1	1.4 ± 0.9^**^

Compared with NS + P group, ^*^P = 0.002, ^#^*P* = 0.039, ^**^P = 0.003

Normally distributed data were expressed as mean ± standard deviation and the data that does not conform to the normal distribution were expressed as p_50_ (p_25_, p_75_)

## Discussion

The main finding of this study was that the addition of intravenous of lidocaine to propofol-based sedation regime had significantly reduced the requirement of supplemental propofol after the initial dosage of anesthesia induction. The “unit propofol”, calculated after dividing the total amount of consumed propofol by patients’ weight and the length of anesthesia, was also significantly lower in L + P group than NS + P group.

Our pre-study observation found that an initial induction dose of 1.2 mg kg^− 1^ propofol combined with 2.5 μg sufentanil had satisfied the need for endoscope insertion in the elderly patients, so this standardized combination for anesthesia induction has been applied in this study.

Forster et al. [7] studied the sedation of intravenous lidocaine combined with propofol and ketamine in adult patients aged 18–70 years old during colonoscopy and he found that intravenous lidocaine allowed a significant 50% reduction in propofol consumption. Altermatt et al. [21] observed the effect of intravenous lidocaine in the perioperative period in patients undergoing elective laparoscopic cholecystectomy, and his results indicated that intravenous lidocaine also reduced the propofol requirement. This propofol-sparing effect by lidocaine had been observed especially during the surgical stimulation. In this study, the supplemental amount of propofol, the bolus frequency of supplemental propofol administered after the initial dosage of anesthesia induction, and the calculated “unit propofol” infusion rate during colonoscopy were significantly reduced when intravenous lidocaine was added to propofol-based sedation, which were consistent with previous studies. However, we did not find statistically significant difference in the total amount of propofol consumed for the induction plus supplement between two groups. The possible explanations were:1) generally, the elderly patients demand much less anesthetics than younger adults to achieve the same level of anesthesia as we only gave smaller than average of induction (1.2 mg kg^− 1^) and supplemental doses (only 0.6 mg kg^− 1^) of propofol; 2) the colonoscopy itself is a pretty short procedure and in many occasions, it can be completed just by a single induction dose of propofol with sufentanyl, which led to less propofol consumption. Those factors would narrow the numerical differences between groups during statistical analysis.

Although there were some studies using combined intravenous lidocaine and propofol for sedation in adult patients undergoing colonoscopy or surgical operation [7, 21], the hemodynamic profiles were rarely reported in this setting, especially in the elderly patients. In this study, we explored the propofol-sparing effect of lidocaine and closely observed the hemodynamic effects when intravenous lidocaine was given in combination with propofol. The results demonstrated that the noninvasive blood pressure and heart rate showed no difference between two groups at all sequential monitoring points, nor did SpO_2._ Those results were highly expected because the patients recruited in our study were relatively healthy (ASA I and II) even though they were aged, and they only received light doses of propofol for induction and sedation maintenance.

Pain felt by patients at peripheral intravenous site on injection of propofol is common in reality. Studies have showed that either pretreatment with lidocaine or mixed lidocaine with propofol would reduce the pain incidence of propofol injection significantly [22, 23]. Our study results showed lower rate of propofol injection pain in L + P group (11/40) than NS + P group (18/40) even there was no difference statistically. Most previous studies were focusing on the younger adults or pediatric patients [23] while ours was done in elder population (> 65 year old). Whether the blunted sensitivity to noxious pain stimulation developed in aging people would be a very interesting subject in our future study.

There were limitations for this study. Firstly, objective indicators, such as BIS or EEG monitoring were not measured during the procedure, so there might be slight variation in judgment of the sedation depth among the individual patients when MOAA/S score, the subjective observation technique was applied. We tried to standardize the personal assessment skill to a single anesthesiologist who was totally blinded to patients’ grouping to minimize the potential bias. Secondly, this study only recruited relatively healthy (ASA I or II) patients and did not extend to more sicker patients (ASA III or IV) who are definitely more vulnerable to the anesthetics.

## Conclusions

In summary, the addition of intravenous lidocaine to propofol sedation during colonoscopy led significant reduction in both of the supplemented propofol and the frequency of supplemental boluses of propofol without compromises of hemodynamic and respiratory profiles.

## Data Availability

The datasets used and/or analysed during the current study available from the corresponding author on reasonable request.

## Abbreviation

MOAA/S scale: Modified Observer’s Assessment of Alertness/Sedation Scale
